# Combination of berberine and evodiamine inhibits intestinal cholesterol absorption in high fat diet induced hyperlipidemic rats

**DOI:** 10.1186/s12944-017-0628-x

**Published:** 2017-12-11

**Authors:** Xin Zhou, Fengying Ren, Hong Wei, Liyun Liu, Tao Shen, Shijun Xu, Jiangping Wei, Jiayue Ren, Hengfan Ni

**Affiliations:** 1 School of Basic Medicine, Chengdu University of Traditional Chinese Medicine, Chengdu, 611137 China; 2 Antibiotics Research and Re-evaluation Key Laboratory of Sichuan Province, Sichuan Industrial Institute of Antibiotics, Chengdu University, Chengdu, 610052 China; 3 School of International Education, Chengdu University of Traditional Chinese Medicine, Chengdu, 611137 China; 4 School of Pharmacy, Chengdu University of Traditional Chinese Medicine, Chengdu, 611137 China

**Keywords:** Berberine, Evodiamine, Hyperlipidemia, Cholesterol absorption, NPC1L1, ACAT2, ApoB48

## Abstract

**Background:**

Hyperlipidemia characterized of elevated serum lipid levels is a prevalent disease frequently resulting in cardiovascular disease (CVD). Berberine and evodiamine are herbal products of traditional Chinese herb *Coptis chinensis* and *Evodia rutaecarpa*, which are indicated to exert regulation of lipid metabolism. Therefore, the objective of this study was to investigate the lipid-lowering effect of berberine and evodiamine combination in hyperlipidemic rats.

**Method:**

The rat model of hyperlipidemia was established by providing high-fat-diet (HFD) for 4 weeks. Berberine (BB), evodiamine (EV), and their combination (BB + EV) were orally administered to HFD induced rats for 4 weeks. Body weight, food utilization, histopathology of liver tissues, lipid profiles of serum and liver were measured. Gas chromatography (GC) analysis was applied to examine the level of plasma total cholesterol and ß- Sitosterol (BS) to estimate cholesterol absorption activity. Furthermore, intestinal NPC1L1, ACAT2, and ApoB48 protein expressions were evaluated by immunohistochemical assay.

**Result:**

According to the results, decreased levels of serum cholesterol (TC), triglycerides (TG), low density lipoprotein-cholesterol (LDL-C), as well as hepatic TC were showed in hyperlipidemic rats treated by combination of berberine and evodiamine. GC analysis indicated that the elevated plasma BS was significantly ameliorated by BB, EV, and BB + EV. In addition, immunohistochemical analysis revealed that BB + EV treatment down-regulated the expressions of intestinal NPC1L1 and ACAT2, and ApoB48 in HFD induced rats.

**Conclusion:**

Based on the above results, combination of berberine and evodiamine exerted a promising preventive effect on hyperlipidemia, partially through inhibiting intestinal absorption of cholesterol.

## Background

Hyperlipidemia is an abnormality of lipid metabolism, characterized by an elevation of total cholesterol (TC), triglyceride (TG), and low density lipoprotein-cholesterol (LDL-C), and/or a decreasing of high density lipoprotein-cholesterol (HDL-C) in circulating levels [1]. Cholesterol homeostasis plays a notable role in maintaining blood lipid level. A large number of studies have demonstrated that disruption of cholesterol metabolism is a high risk of cardiovascular disease (CVD) [2, 3]. Absorption of cholesterol in intestine is a significant physiological process which counts for the transportation of chylomicron cholesterol to liver, involved in maintaining lipid homeostasis [4]. In view of the close relationship between cholesterol absorption and lipid metabolism, cholesterol absorption pathway has been recognized as an important pharmacological intervention for the therapeutic target of hyperlipidemia [5]. Thus, the prevention of hyperlipidemia can be achieved by reducing the intestinal absorption of cholesterol [6].

Berberine is an alkaloid found in the roots of *Coptidis Rhizoma* which is a traditional Chinese medicine known as Huanglian. Berberine has been testified to exert various biological effects including anti-inflammatory, anti-cancer,anti-microbial, and anti-diabetic activities [7–10]. Extensive numbers of studies have confirmed berberine with its hypoglycemic and hypolipidemic effect in clinical and experimental researches [10, 11]. It has been reported that administration with berberine by gavaging for 8 weeks could reduce serum total cholesterol level by 29%–33% through inhibiting the intestinal absorption in high fat diet induced rats [12]. Evodiamine, the major bioactive component found in *Evodia rutaecarpa*, possess beneficial pharmacological effects involving anti-cancer, anti-inflammation, anti-obesity and anti-diabetes effects [13–17]. Previous research has reported that evodiamine could influence lipid metabolism through regulation of the expressions of peroxisome proliferator-activated receptor-g (PPARg) in mouse adipose and liver tissues [18]. Furthermore, Chao DC et al. found that co-administration of berberine and evodiamine could suppress tumor promotion primarily through AP-1 and/or NF-κB pathways in HepG2 cells [19]. Previous research of our laboratory has found that berberine concomitant with evodiamine had ameliorative effect in high fat diet induced rats. However, the effect of the combination of berberine and evodiamine on modulation of cholesterol absorption has not been experimentally researched. As for the lipid-regulated effects of the two drugs, exploration of the combination of berberine and evodiamine on modulation of lipid metabolism through cholesterol absorption would provide interesting results.

Therefore, the present study was undertaken to determine whether the combination of berberine and evodiamine exerted their protective effect on hyperlipidemia induced by high-fat diet(HFD). Moreover, the possible anti-hyperlipidemic mechanisms involving inhibiting the cholesterol absorption would also be discussed.

## Methods

### Chemicals and reagents

Berberine was purchased from Kangdele Pharmaceutical Enterprise (Chengdu, China), while Evodiamine was purchased from Chengdu ruiteen science and Technology Co., Ltd., and dissolved in 0.5%(*v*/v) Carboxymethyl cellulose. Ezetimibe was supplied by Pfizer Pharmaceuticals Ltd. Commercial kits for testing plasma total cholesterol (TC), triglyceride (TG), hepatic TC, and hepatic TG was supplied by Applygen T Gene Technology Co. Ltd. The kits for measurement of low-density lipoprotein cholesterol (LDL-C) and high-density lipoprotein cholesterol (HDL-C) were supplied by Jiancheng Bioengineering Institute (Nanjing, Jiangsu, China).

### Animals

A total of 60 male Sprague-Dawley (SD) rats (10 weeks old, body weight: 200 ± 20 g) were obtained from Chengdu Dashuo experimental animal Co. Ltd. (Certificate no. SCXK (Sichuan) 2013–24). All animals were housed 5 per cage under the controlled conditions (temperature 22 ± 2 °C, relative humidity 50% ± 5%, and 12 h light/dark cycle), and had access to food and water ad libitum. All the rats were fed with the standard solid food as acclimatization for 7 days prior to the experiments. All of the animal protocols were performed in strict accordance with the guidelines for the care and use of laboratory animals established by the Animal Ethical Committee of Chengdu University of Traditional Chinese Medicine (CDUTCM). All surgery was executed under sodium pentobarbital anesthesia, which kept the experimental damage at a minimum.

### Experimental design and supplementation

The 60 rats were randomly assigned to two groups: a normal control group (CON) of 10 was fed with a normal laboratory diet, while 50 rats were fed with HFD for 4 weeks to induce hyperlipidemia. The composition of HFD followed the method of Zhang, Q and co-workers, which contained a normal diet supplemented with 1% cholesterol, 10% lard, 5% yolk powder, 0.2% propylthiouracil and 1% sodium tauroglycocholate [20]. After 4 weeks of feeding, the 50 HFD induced rats were subdivided into five groups: a group of 10 (HFD) was treated with sodium carboxymethyl cellulose (CMC; 0.5%); the other four groups were treated with ezetimibe (EZ) (10 mg/kg b.w.), berberine (BB) (72.6 mg/kg b.w.), evodiamine (EV) (16.6 mg/kg b.w.), and combination of berberine and evodiamine (BB + EV) (89.2 mg/kg b.w.). The berberine, evodiamine, and their combination were dissolved in 0.5% CMC. These compounds were administrated to rats by gavage at a dose of 10 ml/kg body weight for four weeks fed with HFD.

### Sample collection and preparation

During the experiment of 8 weeks, blood sample of each rat was collected from the tail vein under fasting conditions once a week at 9:00 AM, and animal weight of the rats was measured weekly. Serum was separated by centrifugation for 5 min at 3500 rpm, stored at −80 °C. At the end of the experimental period, the animals were fasted for 12 h and then sacrificed after 10% chloral hydrate anesthesia. Cardiac puncture was instantly executed to collect the blood. Livers were excised, weighted and divided into two parts: One part was fixed in 10% neutral buffered formalin for the histopathological detections, the other part was stored at −80 °C for further biochemical studies. Small intestine was resected in a small section (about 8–10 cm) from each rat, stored at −80 °C for immunohistochemical analysis. 10% *w*/*v* homogenate compound was prepared with 0.1 g of liver tissue in 1 ml of 0.1 M phosphate buffer (pH 7.4), then centrifuged at 4000 rpm for 10 min, kept frozen at −80 °C for biochemical assays.

### Lipid profile analysis

The levels of TC, TG, LDL-C, and HDL-C in rat serum were measured by commercial reagent kits respectively, according to the standards and protocols provided by the manufacturer. The liver tissue was homogenized with phosphate buffer (pH 7.4) as density of 10% and centrifuged at 4000 rpm for 10 min. The levels of total cholesterol and triglycerides in the liver were determined by enzymatic-colorimetric assay kits respectively. All the procedures were following the instructions offered by the kits.

### Histopathological examination

The liver tissues were fixed in 10% neutral paraformaldehyde overnight. Then, the samples were dehydrated through graded ethanol series, made transparent with xylene, embedded with paraffin. The sections were cut into 4–8 μm slices and stained with hematoxylin and eosin staining (H&E staining). There were 5 slices for each animal used for pathological observation. The sections were observed at 400× magnification under light microscope.

### Gas chromatography analysis

Concentrations of ß-sitosterol (BS) and cholesterol in serum were measured using Gas Chromatograph (GC) technique. The plasma samples preparation were operated by mixing 0.1 ml of serum sample with 0.1 ml of internal standard, 1.0 ml of absolute ethyl alcohol, and 1.0 mL of 8.9 mol/L KOH. The solution was mixed for 15 s and saponified in 60 °C for 60 min, extracted with hexyl hydride. The supernatant was transferred to a 25 mL volumetric flask and evaporated to dryness at 40 °C. 600 μl of silylating reagent (TMCS / HMDS/ Pyridine (3:1:9, *v*/v)) were added to the solution to catalyze the reaction which proceeded for 30 min at 60 °C. After evaporated to dryness, the reaction system was added with 1 ml of hexyl hydride. Then, the solution was mixed and centrifuged at 12000×g at 4 °C for 1 min. Liquid supernatant was were separated for Gas chromatography analysis. One microliter of prepared sample was injected into a 7890A Gas Chromatograph (Agilent, USA). Chromatographic separation was carried on a HP-5 quartz capillary column (30 m × 0.32 mm × 0.25 μm), and high purity nitrogen (99.99%) was applied as carrier gas. The temperature of Hydrogen flame ionization detector (FID) was set to 290 °C. The temperature and pressure of injection port were controlled at 290 °C and 18 psi, respectively, while no spit mode administrated. The initial temperature of column was kept at 180 °C for 3 min. The temperature was following increased up to 260 °C at a rate of 15 °C/min, and then elevated to 300 °C at a rate of 5 °C/min and held for 10 min. Plant sterols were identified by the gas chromatography peaks in contrast with the standards, which were measued as mg fatty acid per ml plasma volume.

### Immunohistochemistry assay

The intestinal samples were washed by PBS rapidly and fixed with 4% paraformaldehyde overnight at 4 °C. With accomplishment of 4 μm paraffin slices, the sections were deparaffinized with xylene and rehydrated through gradient ethanol, treated with 3% hydrogen peroxide methanol for 10 min which were used to inactivate endogenous peroxidase. Washed with PBS for 3 times, tissue sections were subjected to 10 mM sodium citrate buffer (pH 6.0) and heated in microwave oven with middle power for 10 min. After blocked in goat serum for 20 min at room temperature, the sections were incubated with primary polyclonal rabbit anti- ACAT2, anti- NPC1L1, or anti- Apo B, respectively, at 4 °C for one night. Subsequently, sections were incubated in goat anti-rabbit IgG for 30 min at 37 °C before permeabilization in PBS. Diaminobenzidine was applied for chromogenic reaction for 2 min at the room temperature. Then slides were treated with Light Mayer hematoxylin for counterstaining, and then dehydrated by graded concentration of ethanol, finally covered with Neutral Canada gum. Images were captured using an BA400 Digital confocal microscope. The integrated optical density (IOD) of ACAT2, NPC1L1, and Apo B expressions were measured by Image-Pro Plus 6.0 software (Media Cybernetics, Siver Spring, MD, U.S.A.), meanwhile the images were managed with Adobe Photoshop 8.0.

### Statistical analysis

All experimental data were expressed as mean ± standard deviation (S.D.). Statistical analyses were evaluated by one-way analysis of variance (ANOVA) and two-tailed Student’s t tests with SPSS19.0 software. A level of *P*-value <0.05 was considered statistically significant. All the figures were carried out by Graph Pad Prism 6.0 software.

## Results

### Animal and organ weights

**Fig. 1:**
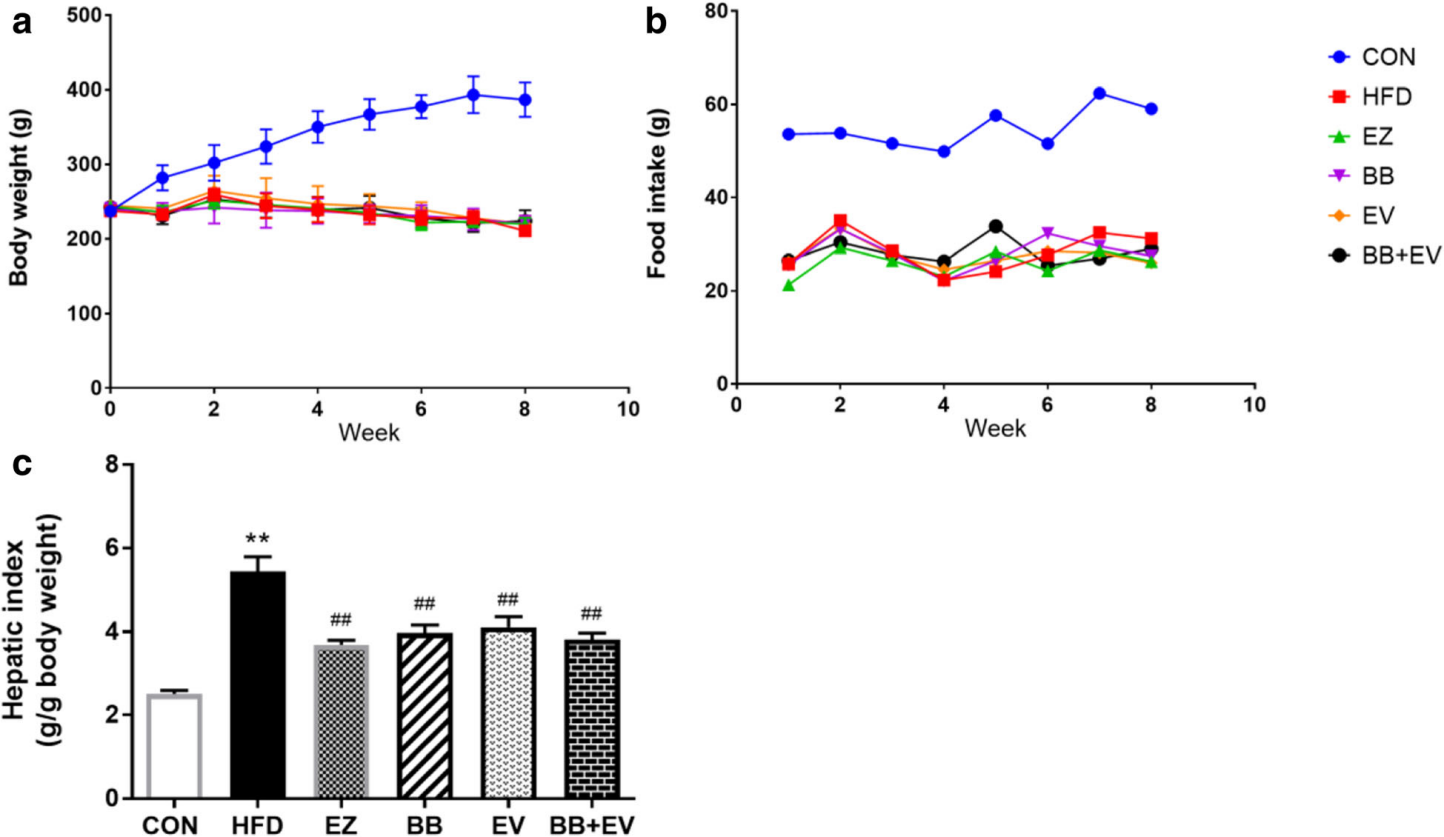
Body weights (**a**) and food intake (**b**) of each group in week 0, 2, 4, 6, and 8; liver weight index (liver weight/body weight) (**c**) of each group in week 8. Data are represented as mean ± SD with 10 samples each group. *P < 0.05,**P < 0.01 compared with Control; #*P* < 0.05,##*P* < 0.01 compared with Model (ANOVA with Dunnett’s post-hoc test)

During the experimental period, body weight and food intake declined remarkably in the HFD-fed groups compared with those in CON group (*P* < 0.01). However, no significant difference was showed among the groups administrated by different treatments in body weight and food intake (*P* > 0.05) (Fig. 1a and b). Liver weight index was detected as the ratio of liver weight to body weight to properly compare individual variation on liver pathology. Compared with CON, HFD group showed remarkable increase on liver weight index, which were significantly reduced by BB, EV, and BB + EV (*P* < 0.01) (Fig. 1c).

### Serum lipid profiles

**Fig. 2:**
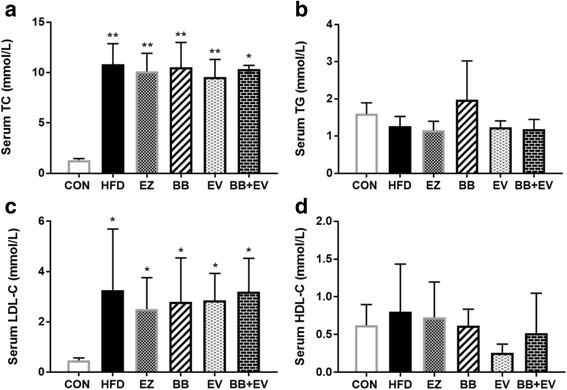
Serum TC (**a**), serum TG (**b**), serum LDL-C (**c**), and serum HDL-C (**d**) in hyperlipidemic rats in week 4. Data are represented as mean ± SD with 10 samples each group. *P < 0.05,**P < 0.01 compared with Control; #*P* < 0.05,##*P* < 0.01 compared with Model (ANOVA with Dunnett’s post-hoc test)

**Fig. 3:**
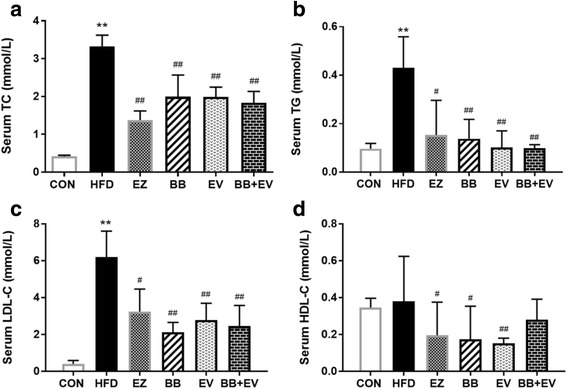
Serum TC (**a**), serum TG (**b**), serum LDL-C (**c**), and serum HDL-C (**d**) in hyperlipidemic rats in week 8. Data are represented as mean ± SD with 10 samples each group. *P < 0.05,**P < 0.01 compared with Control; #*P* < 0.05,##*P* < 0.01 compared with Model (ANOVA with Dunnett’s post-hoc test)

The level of serum TC, TG, HDL-C, and LDL-C in hyperlipidemic rats at 4th and 8th week were showed in Fig. 2-3. By comparison with CON group, meaningful elevation of serum TC and LDL-C was observed in the HFD-fed rats in week 4 (*P* < 0.05 or *P* < 0.01) (Fig. 2a, c). But the concentration of serum TG and HDL-C did not show significant difference when rats were fed with HFD (*P* > 0.05) (Fig. 2b, d).

After the continuation of HFD supplementation and treatment by tested drugs for 4 weeks, remarkable reductions were observed in hyperlipidemic rats treated by different drugs in week 8 (Fig.3).The serum levels of TC, TG, and LDL-C were significantly decreased in group BB, EV, and BB + EV compared with those in HFD group in week 8 (*P* < 0.05 or *P* < 0.01) (Fig. 3a, b, c). Only plasma HDL-C concentration showed a difference between HFD group and other drug treated groups. The administration of BB and EV for 4 weeks resulted in notable decreases in serum HDL-C levels (*P* < 0.05 or *P* < 0.01), while there was no statistical significance on LDL-C level when compared with the HFD group (Fig. 3d).

### Lipid content of the liver

**Fig. 4:**
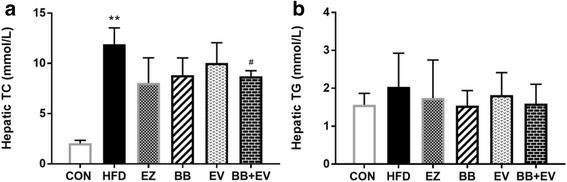
Hepatic TC (**a**) and hepatic TG (**b**) content of hyperlipidemic rat in week 8. Data are represented as mean ± SD with 10 samples each group. *P < 0.05,**P < 0.01 compared with Control; #*P* < 0.05,##*P* < 0.01 compared with Model (ANOVA with Dunnett’s post-hoc test)

Hepatic cholesterol and triglycerides were measured at the end of the 8-weeks experimental period to analyze the effect of BB and EV on tissue lipid content. Compared with CON group, the level of hepatic TC were significantly increased in hyperlipidemic rats induced by HFD (*P* < 0.01). Hepatic TC content in BB + EV group were remarkable decreased by 26% compared with the HFD group (*P* < 0.05). But no noticeably significant was showed when rats were administrated only with BB or EV (Fig. 4a). Treatment with BB and BB + EV tended to reduce hepatic triglycerides levels, although just in the edge of significance (*P* > 0.05) (Fig. 4b).

### Histological analyses of the liver

**Fig. 5:**
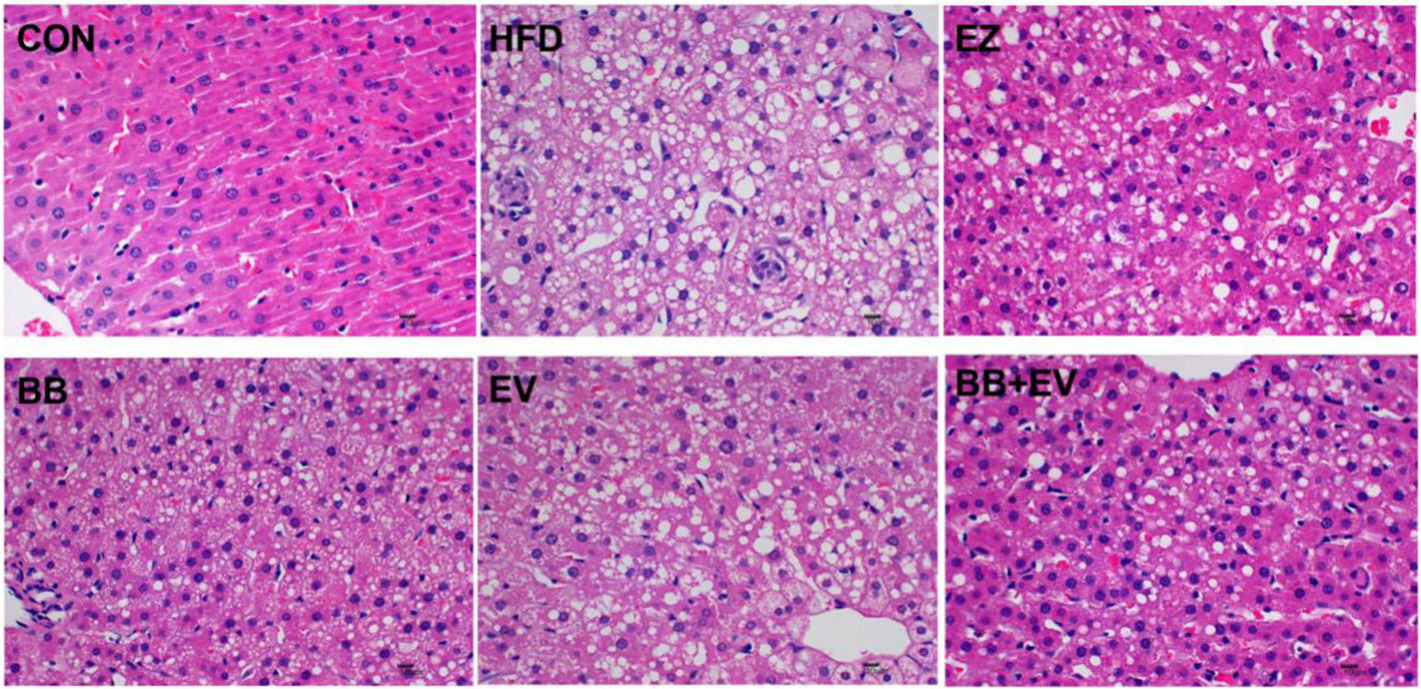
Effect of berberine and evodiamine co-administration on the pathology of liver tissues in hyperlipidemic rats. Specimens were photographed by light microscopy. (H and E staining, magnification: ×400, *n* = 6)

After supplementation of HFD for eight weeks, liver structures and the degree of lipids accumulation were characterized by hematoxylin-eosin (H&E) staining on liver tissues of rats with hyperlipidemia. As showed in Fig. 5, the rats in CON group showed a well- structured liver. HFD rats manifested pathological changes of the liver manifested swollen hepatocytes coupled with hepatocytes steatosis in the form of increasing degree of vacuolar degeneration. By comparison of HFD group, these changes could be obviously alleviated by EZ, BB, and BB + EV (Fig. 5). HE staining indicated that fatty liver changes accompanied with neutrophils infiltrating into acini area were resulted from HFD, which could be obviously inhibited by EZ, BB, and BB + EV.

### Absorption markers of cholesterol

**Fig. 6:**
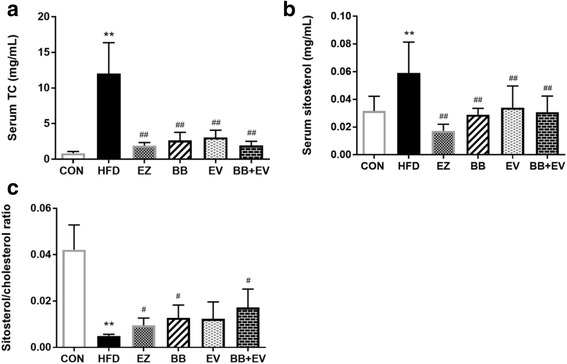
Serum total cholesterol (**a**), serum sistosterol (**b**), and the ratio of sistosterol by cholesterol (**c**) measured by gas chromatography in hyperlipidemic rat. Data are represented as mean ± SD with 10 samples each group. *P < 0.05,**P < 0.01 compared with Control; #*P* < 0.05,##*P* < 0.01 compared with Model (ANOVA with Dunnett’s post-hoc test)

Plasma concentrations of BS and cholesterol measured by GC analysis in hyperlipidemic rats were showed in Fig. 6. By the continuation of 8 weeks supplementation with the HFD, serum levels of BS and cholesterol were significantly elevated in the HFD group compared with the CON group (*P* < 0.01). After 4 weeks of treatment with the therapies, serum contents of BS and cholesterol were rapidly decreased in group BB, EV, and BB + EV (*P* < 0.01) (Fig. 6a, b). Additionally, the ratio of BS to cholesterol developed a significant decrease in HFD group, which was alleviated by BB and BB + EV (*P* < 0.05) (Fig. 6c).

### Immunohistochemistry

**Fig. 7:**
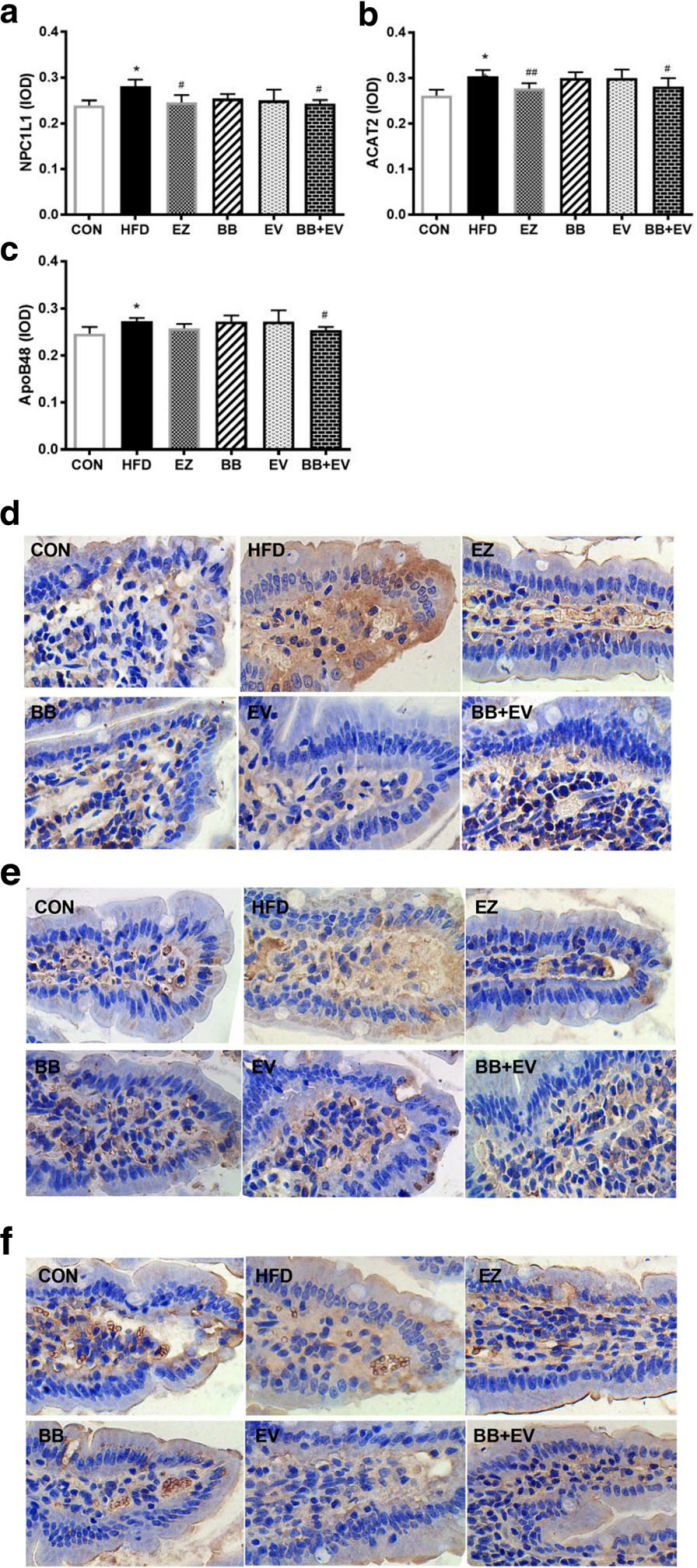
Integrated optical density (IOD) of NPC1L1(**a**), ACAT2 (**b**), and ApoB48 (**c**) in HFD induced hyperlipidemic rats (*n* = 6, mean ± SD). *P < 0.05,**P < 0.01 compared with Control; #*P* < 0.05,##*P* < 0.01 compared with Model (ANOVA with Dunnett’s post-hoc test). Histopathological examination of NPC1L1 (**d**), ACAT2 (**e**), and ApoB48 (**f**) in intestine tissue of hyperlipidemic rats in different experiment groups (×400 magnifications, *n* = 6)

The immunohistochemical staining of intestine tissues in hyperlipidemic rats were shown in Fig. 7. Positive cells expressed as brown or yellow particle, while the negative cells showed blue stained nucleus. ACAT2, NPC1L1, and ApoB48 positive reactions were mainly distributed in the small intestine mucosa epithelium according to the staining results (Fig. 7d, e, and f). Meanwhile, the integrated optical density (IOD) of ACbAT2, NPC1L1, and ApoB48 expressions were calculated to evaluate the effect of berberine combined with evodiamine on cholesterol absorption in intestine. Compared with the CON group, HFD increased the expressions of ACAT2, NPC1L1, and ApoB48 significantly. Furthermore, berberine combined with evodiamine could remarkably decrease the expressions of intestine ACAT2, NPC1L1, and ApoB48 compared with HFD group (*P* < 0.05) (Fig. 7a, b, and c). Treatment with BB alone had the trend to decrease the expression of intestinal NPC1L1 protein, even though barely failed to attain statistical significance (*P* > 0.05) (Fig. 7a).

## Discussion

Hyperlipidemia presents as a principal factor of accelerating the progression of Cardiovascular disease (CVD) which results in morbidity and mortality in developed countries [21]. Treatments of lipid lowering is well-recognized as a notable strategy by all the health care organizations around the world for prevention of CVD [22]. A large number of natural plant products found in traditional Chinese medicine (TCM) have been proved to possess lipid-lowing activities with fewer side effects, which draw great attention worldwide [23]. These findings have enhanced the searching for the alternative drugs with fewer adverse effects to prevent the risk of CVD. The combination of berberine and evodiamine is from a two-herb Chinese medicinal formula composed of *Coptis chinensis* and *Evodia rutaecarpa*, which has been widely used to treat multiple diseases in terms of different ratios [24]. Berberine is the active ingredient of the traditional herbal *Coptidis Rhizoma* which has been investigated in many directions, including anti-inflammatory, anti-cancer,anti-microbial, as well as cardiovascular fields [7–10]. Evodiamine is a naturally indoloquinazoline alkaloid isolated from *Evodia rutaecarpa*, attracting close attentions with its anti-cancer, anti-inflammation, and anti-obesity effects [13–17]. Many researches have indicated that both of berberine and evodiamine exerted on regulation of lipid metabolism [18, 25], but whether they have a synergistic effect on preventing hyperlipidemia when combined together remains completely unclear.

In the present study, the effect of the berberine combined with evodiamine was compared to the effects of a single herb on the parameters of hyperlipidemia in HFD-fed rats. The current study demonstrated that berberine, evodiamine, and their combinations could obviously reduce the serum lipid hyperlipidemic rats induced by HFD. Moreover, the combination of berberine and evodiamine notably decreased the content of TC in serum and liver, which showed best lipid-lowing effect compared with the either drug alone. The results appeared to be show a synergistic action of berberine and evodiamine as the effect on serum TC reduction. The underlying mechanism was exerted by the characterization of key proteins involved in the process of cholesterol absorption.

Diet habits play a significant role in the development of cholesterol metabolism. It is believed that the consumption of high fat diet which contains of saturated fats is the major risk factor for development of obesity and metabolic syndrome [26]. In this study, the hyperlipidemic animal model was successfully established in rats by the induction of high-fat, high-cholesterol diet, as evidenced by increased lipid parameters in serum and liver. In our study, a dramatic increase in serum lipid levels of TC, TG, LDL-C, and hepatic TC levels was observed in hyperlipidemic rats induced with HFD in 4 weeks (Fig. 2). After administration with berberine, evodiamine, and their combination, the elevated serum levels of TC, TG, and LDL-C were significantly decreased at eight week (Fig. 3). Meanwhile, cholesterol content in liver was obviously decreased after the supplementation of BB + EV. Notably, treatment with BB + EV showed the best effect on normalizing the serum TC, TG, and hepatic TC concentrations in 8 weeks, which appeared superior to either herb alone. However, BB and BB + EV did not develop a significant effect on body weight compared with HFD group (Fig. 1a, b), which agreed with the study of Yanwen Wang et al. [12], who found that berberine did not show a significant effect on body weight. Berberine was conformed to possess obvious cholesterol-lowering effect on serum TC, LDL-C, or non HDL cholesterol (non HDL-C) in plenty of researches [12, 27–29]. Evodiamine was reported to have effect on lipid metabolism by regulation the key genes in fat synthesis [18]. The reductions of liver cholesterol might be a result of decreased cholesterol input arise from the inhibition of intestinal cholesterol absorption [30]. Our research showed that the combination of berberine and evodiamine had the best lipid-lowing effect on lipid profile in serum and liver, compared to either drug alone. Therefore, we speculate that the berberine combined with evodiamine had a synergistic effect on reducing cholesterol in the plasma and liver by inhibiting intestinal cholesterol absorption.

Large numbers of studies have demonstrated the hypolipiemic properties of berberine and evodiamine by animal and clinical researches. Kong et al. [31] has been reported that 1000 mg/day berberine for three months could significantly lower total cholesterol and LDL-cholesterol by 29% and 25%. Another study has showed that evodiamine as a supplement comprising 0.02, 0.04, and 0.06% of the diet fed to mice for 4 weeks could influence lipid metabolism in time and dose-dependent manners [18]. However, minimal information on the adverse drug reactions of berberine and evodiamine supplementation is available. Some studies have demonstrated that the maternal toxicity lowest observed adverse effect levels of berberine were 531 mg/kg/day and 841 mg/kg/day in rats and mice [32], and berberine could present certain side effects in hypervagotonic people [33]. The incidence of toxic adverse effect is connected with the doses of berberine, which showed that the dose of berberine increase lead to the rising risk of toxic side effect [34]. In our current study, the doses of berberine and evodiamine were 72.6 mg/kg and 16.6 mg/kg, which were equal to 5 g/day of the human dose, much lower than that used in previous studies. Whether the supplementation of berberine combined with evodiamine has any side effects for a long time as a treatment on hyperlipidemia needs a further study in the future.

Hyperlipidmia is characterized as the increased serum lipid index especially serum total cholesterol and LDL cholesterol levels, which results in coronary morbidity and mortality. The previous researches suggested that the ratios of serum cholestanol and plant sterols to cholesterol were the markers of cholesterol absorption, which reliably depicted the cholesterol absorption efficiency. Non-cholesterol sterols which include cholestanol and plant sterols, are regarded to be significant markers of cholesterol absorption [35]. Thongtang et al. [36] found that the ratio of ß-sitosterol to total cholesterol was positively related to absorption activity of cholesterol. In this study, we found that supplementation with HFD increased the level of ß-sitosterol and ß-sitosterol/cholesterol ratio, which were attenuated by berberine, evodiamine, and their combination, suggesting that berberine, evodiamine, and their combination could successfully decrease the markers of cholesterol absorption, ultimately inhibiting absorption of cholesterol. Notably, treatment with the combination of berberine and evodiamine showed remarkable reduction in ß-sitosterol by 48.2% compared with the HFD group. Although berberine alone also inhibited the cholesterol absorption marker, this effect was intensified when it was combined with evodiamine. Therefore, we hypothesized that the hyperlipidemic ability of co-administration of berberine and evodiamine may be associated with their effects on cholesterol absorption.

Absorption of cholesterol is a significant physiological process involved in maintaining lipid homeostasis [4]. Cholesterol absorption occurs mainly in the small intestine and proximal jejunum,which is regulated by a number of proteins. Niemann-Pick C1 like1 (NPC1L1) protein and Acyl-CoA cholesterol acyltransferase 2 (ACAT2) are essential factors on regulating the intestinal absorption of cholesterol. Cholesterol absorption starts with cholesterol uptake by enterocyte. Apart from the passive penetration, free cholesterol which is hydrolyzed from dietary cholesterol esters and biliary sources are transported actively through NPC1L1 from the lumen into enterocytes [37]. Subsequently, free cholesterol is converted to cholesteryl ester (CE), packed into chylomicrons (CM) and then transferring into the lymphatics. This process is catalyzed by ACAT2, which is a key cholesterol esterifying enzyme for chylomicrons biosynthesis from cholesteryl esters [38]. After the general circulation, cholesterol is ultimately delivered to the liver as the form of CM. Our study showed that the co-administration of berberine and evodiamine favorably inhibit the expressions of NPC1L1 and ACAT2 accompany by the reductions of serum cholesterol levels, which suggested that the hyperlipidemic effect of the combination might be attributed to the down-regulation of NPC1L1 and ACAT2 in HFD-fed rats. Elimination of excessive cholesterol contained mainly two ways, which included firstly, combining the intracellular cholesterol into cholesterol esters in a way activation of ACAT2. Second, excess cholesterol was converted to bile acids in the liver, finally eliminated into feces [39]. The second possible mechanism for the cholesterol-lowering activity of berberine and evodiamine supplementation was mediated by increasing the bile acid synthesis. We plan to investigate the effect of combination of berberine and evodiamine on bile acid profile in the future study.

Apolipoprotein B is an important plasma particle which is the formation of chylomicrons and the other cholesterol transport molecules as low-density lipoprotein (LDL) and very-low-density lipoprotein (VLDL) for cholesterol transportation between liver and peripheral tissues [40]. ApoB48 is the transport molecules essential for CM, responsible for packing the dietary fats to form CMs for transportation [41]. In fact, ApoB48 was accepted as a vital element involved in lipid metabolism, even a risk factor for CVD [42]. In line with previous study, the current study found that the expression of ApoB48 in intestine was importantly elevated in HFD group by comparison with the CON group. Interestingly, berberine combined with evodiamine significantly decreased intestinal ApoB48 expression in HFD-fed rats. These results indicated that the combination of berberine and evodiamine exhibited the lipid-lowing effect partly on inhibiting the intestinal absorption of dietary fat by reducing ApoB48 overproduction.

## Conclusion

In conclusion, our study indicated that berberine, evodiamine, and their co-administration can lower blood cholesterol levels in hyperlipidemic rat induced by high fat diet. Additionally, evodiamine functions as an enhancer of berberine to promote the lipid-lowing effect on serum cholesterol in HFD-induced rats, and the potential mechanism appears to involve inhibiting the expression of ACAT2, NPC1L1, and ApoB48 to depress the cholesterol absorption. Thus, our outcomes demonstrated that the combination of berberine and evodiamine possessed potential lipid-decreasing effect, and further researches on berberine combined with evodiamine deserve to be carried out to provide new insight into maintaining healthy lipid profiles.

## Abbreviations

ACAT2: Acyl-CoA cholesterol acyltransferase 2
ApoB48: Apolipoprotein B48.
BB: Berberine
BS: ß- Sitostero
CE: Cholesteryl ester
CM: Chylomicrons
CMC: Sodium carboxymethyl cellulose
CON: Control
CVD: Cardiovascular disease
EV: Evodiamine
EZ: Ezetimibe
FID: Flame ionization detector
GC: Gas chromatography
HDL-C: High density lipoprotein-cholesterol
HFD: High fat diet
HMDS: Hexamethyl Disilazane
LDL-C: Low density lipoprotein-cholesterol
LDLR: Low density lipoprotein receptor
NPC1L1: Niemann-Pick C1 like1
TC: Total cholesterol
TCM: Traditional Chinese Medicine
TG: Triglycerides
TMCS: Tri Methyl Chloro Silane
VLDL: Very-low-density lipoprotein

## References

[1] Xiao C (2016). Pharmacological targeting of the Atherogenic dyslipidemia complex: the next frontier in CVD prevention beyond lowering LDL cholesterol. Diabetes.

[2] Watts GF, Karpe F (2011). Triglycerides and atherogenic dyslipidaemia: extending treatment beyond statins in the high-risk cardiovascular patient. Heart.

[3] Okerson T, et al. Effect of 2013 ACC/AHA blood cholesterol guidelines on statin treatment patterns and low-density lipoprotein cholesterol in atherosclerotic cardiovascular disease patients. J Am Heart Assoc. 2017;6(3) 10.1161/JAHA.116.004909 PMC5524020 28314797

[4] Li Y (2014). Adipose tissue regulates hepatic cholesterol metabolism via adiponectin. Life Sci.

[5] Rudel LL, Lee RG, Parini P (2005). ACAT2 is a target for treatment of coronary heart disease associated with hypercholesterolemia. Arterioscler Thromb Vasc Biol.

[6] Gylling H (2004). Polymorphisms in the ABCG5 and ABCG8 genes associate with cholesterol absorption and insulin sensitivity. J Lipid Res.

[7] Choi BH (2006). Berberine reduces the expression of adipogenic enzymes and inflammatory molecules of 3T3-L1 adipocyte. Exp Mol Med.

[8] Ma W (2017). Berberine inhibits the proliferation and migration of breast cancer ZR-75-30 cells by targeting Ephrin-B2. Phytomedicine.

[9] Wang X (2009). Effect of berberine on Staphylococcus Epidermidis biofilm formation. Int J Antimicrob Agents.

[10] Dong H (2012). Berberine in the treatment of type 2 diabetes mellitus: a systemic review and meta-analysis. Evid Based Complement Alternat Med.

[11] Dong H (2013). The effects of berberine on blood lipids: a systemic review and meta-analysis of randomized controlled trials. Planta Med.

[12] Wang Y (2014). Berberine decreases cholesterol levels in rats through multiple mechanisms, including inhibition of cholesterol absorption. Metabolism.

[13] Zhao LC (2015). Evodiamine induces apoptosis and inhibits migration of HCT-116 human colorectal cancer cells. Int J Mol Sci.

[14] Lv Q (2015). Beneficial effects of evodiamine on P2X(4)-mediated inflammatory injury of human umbilical vein endothelial cells due to high glucose. Int Immunopharmacol.

[15] Chiou WF (1992). The vasorelaxant effect of evodiamine in rat isolated mesenteric arteries: mode of action. Eur J Pharmacol.

[16] Hu Y (2010). Inhibitory effect and transcriptional impact of berberine and evodiamine on human white preadipocyte differentiation. Fitoterapia.

[17] Bak EJ (2010). Inhibitory effect of evodiamine alone and in combination with rosiglitazone on in vitro adipocyte differentiation and in vivo obesity related to diabetes. Int J Obes.

[18] Jiang DF (2014). Long-term effects of evodiamine on expressions of lipogenesis and lipolysis genes in mouse adipose and liver tissues. Genet Mol Res.

[19] Chao DC (2011). Inhibitory effects of Zuo-Jin-wan and its alkaloidal ingredients on activator protein 1, nuclear factor-kappaB, and cellular transformation in HepG2 cells. Fitoterapia.

[20] Zhang Q (2009). Application of GC/MS-based metabonomic profiling in studying the lipid-regulating effects of Ginkgo Biloba extract on diet-induced hyperlipidemia in rats. Acta Pharmacol Sin.

[21] Wang J (2017). Novel biomarkers for cardiovascular risk prediction. J Geriatr Cardiol.

[22] Nelson RH (2013). Hyperlipidemia as a risk factor for cardiovascular disease. Prim Care.

[23] Li L (2015). Herbal drugs against cardiovascular disease: traditional medicine and modern development. Drug Discov Today.

[24] Qian P, et al. Pharmacokinetics studies of 12 alkaloids in rat plasma after oral Administration of Zuojin and fan-Zuojin Formulas. Molecules. 2017;22(2) 10.3390/molecules22020214 PMC6155683 28146096

[25] Tan HL (2016). Rhizoma Coptidis: A Potential Cardiovascular Protective Agent. Front Pharmacol.

[26] Kanoski SE, Davidson TL (2011). Western diet consumption and cognitive impairment: links to hippocampal dysfunction and obesity. Physiol Behav.

[27] Wang Y (2010). Berberine and plant stanols synergistically inhibit cholesterol absorption in hamsters. Atherosclerosis.

[28] Kim WS (2009). Berberine improves lipid dysregulation in obesity by controlling central and peripheral AMPK activity. Am J Physiol Endocrinol Metab.

[29] Jia X (2008). Co-administration of berberine and plant stanols synergistically reduces plasma cholesterol in rats. Atherosclerosis.

[30] Hayes KC (2002). Free phytosterols effectively reduce plasma and liver cholesterol in gerbils fed cholesterol. J Nutr.

[31] Kong W (2004). Berberine is a novel cholesterol-lowering drug working through a unique mechanism distinct from statins. Nat Med.

[32] Jahnke GD (2006). Developmental toxicity evaluation of berberine in rats and mice. Birth Defects Res B Dev Reprod Toxicol.

[33] Cannillo M (2013). Berberine behind the thriller of marked symptomatic bradycardia. World J Cardiol.

[34] Lan J (2015). Meta-analysis of the effect and safety of berberine in the treatment of type 2 diabetes mellitus, hyperlipemia and hypertension. J Ethnopharmacol.

[35] *<Noncholesterol Sterols and Cholesterol Lowering by.pdf>.*

[36] Thongtang N (2012). Effects of ezetimibe added to statin therapy on markers of cholesterol absorption and synthesis and LDL-C lowering in hyperlipidemic patients. Atherosclerosis.

[37] Altmann SW (2004). Niemann-pick C1 like 1 protein is critical for intestinal cholesterol absorption. Science.

[38] Lee RG (2005). ACAT2 contributes cholesteryl esters to newly secreted VLDL, whereas LCAT adds cholesteryl ester to LDL in mice. J Lipid Res.

[39] Jiang C (2015). Cholesterol-lowering effects and potential mechanisms of different polar extracts from Cyclocarya paliurus leave in hyperlipidemic mice. J Ethnopharmacol.

[40] Caku A (2017). New insights of altered lipid profile in fragile X syndrome. PLoS One.

[41] Klop B, Elte JW, Cabezas MC (2013). Dyslipidemia in obesity: mechanisms and potential targets. Nutrients.

[42] Pal S (2003). Identification of lipoproteins of intestinal origin in human atherosclerotic plaque. Clin Chem Lab Med.

